# CD155-TIGIT/CD96/CD226 immune checkpoint axis interacting with tumor-infiltrating lymphocytes to exhibit diverse prognostic effects on breast cancer: a cohort study

**DOI:** 10.3389/fimmu.2025.1649078

**Published:** 2025-10-16

**Authors:** Xin Ou, Junyu Yin, Feng Shi, Yanjie Zhao, Quan Zhou, Keyu Yuan, Shuzhen Lyu, Jiangping Wu, Yanping Li, Qingkun Song

**Affiliations:** ^1^ Department of Clinical Epidemiology, Beijing Youan Hospital, Capital Medical University, Beijing, China; ^2^ Center of Biobank, Beijing Youan Hospital, Capital Medical University, Beijing, China; ^3^ Department of Pathology, Beijing Shijitan Hospital, Capital Medical University, Beijing, China; ^4^ Department of Medical Oncology, Beijing Shijitan Hospital, Capital Medical University, Beijing, China; ^5^ Department of Pathology, Cancer Hospital, Chinese Academy of Medical Sciences, Peking Union Medical College, Beijing, China; ^6^ Department of Breast Surgery, Beijing Shijitan Hospital, Capital Medical University, Beijing, China

**Keywords:** CD155-TIGIT/CD226/CD96 immune checkpoint molecules, prognosis, breast cancer, tumor cells, tumor-infiltrating lymphocytes, tumor microenvironment

## Abstract

**Background:**

CD155, an immune checkpoint molecule interacted with receptors of TIGIT/CD96/CD226 to exhibit co-inhibitory and co-stimulatory modulation on tumor immune microenvironment. Nevertheless, the exploration of collectively prognostic effect of these four molecules on breast cancer (BC) was limited. This study aimed to investigate the prognosis effect of CD155-TIGIT/CD96/CD226 complex in BC.

**Methods:**

CD155-TIGIT/CD96/CD226 expression was evaluated by immunohistochemistry in tumor microenvironment (TME) by pathological professionals and the associations with clinical characteristics and prognosis were investigated under a cohort study design.

**Results:**

CD155 was detected on TME tumor cells (TC) and TIGIT/CD96/CD226 were detected on both TC and stromal tumor-infiltrated lymphocytes (TILs). The four molecules showed significant correlation with clinicopathological characteristics and prognosis. High CD155 was associated with relapse (HR = 2.21, 95%CI:1.18-4.13) and death (HR = 2.57, 95%CI:1.29-5.10). High expression of CD226 (HR = 1.79, 95%CI:1.03-3.11) and CD96 (HR = 2.65, 95%CI:1.09-6.44) on TC was correlated with high risk of relapse. High expression of TIGIT on TILs was related to poor prognosis of relapse (HR = 2.06, 95%CI:1.02-4.14), while the expression on TC was a protective factor for relapse (HR = 0.45, 95%CI:0.24-0.83) and death (HR = 0.32, 95%CI:0.16-0.66). Additionally, tumoral and stromal expression of these biomarkers interacted with TME infiltration of stromal TILs to exhibit the diverse prognosis effect.

**Conclusion:**

The CD155-CD226/TIGIT/CD96 immune checkpoint complex expressed on both TME TC and TILs, and interacted with TILs to exhibit diverse prognosis effect on BC. The immunotherapy against these checkpoint proteins should check the expression on both TC and TILs and further studies should explore the molecule complex collectively for comprehensive prediction of BC prognosis.

## Introduction

In 2022, there were approximately 2.3 million Breast cancer (BC) cases and 666,000 deaths worldwide, ranking second in cancer burden in incidence and fourth in the leading cause of death (1). Immune checkpoint inhibitors (ICIs), representing a burgeoning immunotherapy strategy, have enhanced the clinical cure rate of BC patients (2). Nevertheless, some patients cannot obtain any clinical benefits from immunotherapy and suffer from disease progression or recurrence (3). 2.8%-15.8% of BC patients treated with ICIs experience cancer progression or developed new lesions (4–6). Exploration of new prognostic biomarkers and therapeutic targets was essential for immunotherapy.

CD155-TIGIT/CD96/CD226, members of the immunoglobulin superfamily, are potential immunotherapy targets. CD155 is an imperative cell adhesion protein and a key regulator of cell-mediated immune responses in the immunoglobulin superfamily, and is regularly upregulated in malignant tumor cells (TC) (7). TIGIT is a co-inhibitory receptor mainly expressed on T cells and NK cells and has been found to be highly upregulated in tumor-infiltrating lymphocytes (TILs) in melanoma and other cancers (7, 8). CD96 is also expressed mainly on immune cells and is increased in acute lymphoblastic leukemia and myelodysplastic syndrome (9). CD226 is a co-stimulant receptor that had been found to be down-regulated in non-small cell lung cancer and sensitive to clinical therapies (10). With CD155 binding, TIGIT/CD96/CD226 transmits inhibition and activation signals to the immune system, and the integrated signals regulate immune functions and affect the anti-tumor immune response (11). These interactive functions indicate that the complex of CD155 with TIGIT/CD96/CD226 should be evaluated collectively to contribute to the development of new immunotherapeutic targets. In addition, clinical and basic studies have reported the anti-tumor responses of immunotherapy against CD155 (12), TIGIT (13), CD96 (14) and CD226 (15), and elucidated the potential of modulating the CD155-TIGIT/CD96/CD226 immune pathway to enhance the anti-tumor immune response.

Currently, clinical studies of CD155-TIGIT/CD96/CD226 in BC have primarily focused on the expression of CD155 and TIGIT; however, research on CD226 and CD96 is limited. Thus, this study aimed to investigate the prognostic value of these four molecules on TC and stromal TILs and provide a reference for BC immunotherapy.

## Materials and methods

### Ethical approvement

All procedures performed in this study involving human participants were in accordance with the ethical standards of the Institutional Review Board of Beijing Shijitan Hospital, Capital Medical University (sjtkyll-1x-2021(108)), as well as the 1964 Helsinki Declaration and its later amendments or comparable ethical standards (16). Given the retrospective and de-identified nature of the study, the aforementioned Institutional Review Board waived the requirement for written informed consent (16).

### Study setting and design

This study was a retrospective cohort study. 227 female patients with a pathological diagnosis of primary BC were recruited from the Department of Breast Surgery, Beijing Shijitan Hospital, Capital Medical University from 2010 to 2018. Inclusion criteria: ① Patients had no diagnosis of pregnancy, lactation, or other malignancies; ②Patients had no experience of neoadjuvant chemotherapy or target therapy; ③Patients under 75 years of age. Exclusion criteria: ①Patients previously received any form of immunotherapy; ②Patients had a diagnosis of autoimmune disease; ③Patients had an Eastern Cooperative Oncology Group (ECOG) score >2; ④Patients had dysfunction of the heart, brain, kidneys, and other vital organs.

### Data collection and definition

The clinical factors included age, pathological diagnosis, estrogen receptor (ER), progesterone receptor (PR), human epidermal growth factor receptor 2 (HER-2) status, tumor histological grade, tumor stage, Ki-67 status, PD-1 and PD-L1 expression status.

The positive expression threshold of ER and PR in immunohistochemistry (IHC) detection was set to 1% TC staining. IHC tests with 3+ staining or positive results in fluorescence *in situ* hybridization (FISH) indicated positive HER-2 expression. By contrast, IHC tests with less than 2+ staining or negative FISH results showed negative expression. Patients with 2+ staining in IHC tests were required to undergo FISH testing. Ki-67 expression was defined as a brown nucleus in BC cells by IHC on 4 μm-thick formalin fixed paraffin-embedded sections. Meanwhile, the Ki-67 index was calculated as the proportion of BC cells expressing Ki-67 within the hot-spot area. The hot-spot area was determined under a low-power field, and an index≥14% was defined as a high expression of Ki-67. Molecular subtypes were defined as Luminal A (HER-2 negative, PR/ER positive, Ki-67 low expression), Luminal B (HER-2 negative, PR/ER positive, Ki-67 high expression), TNBC (HER-2 negative, ER negative, PR-negative, Ki-67 arbitrary), and HER-2 overexpression (HER-2 positive, Ki-67 arbitrary). Tumor histological grading was performed using the Nottingham grading system, integrating the proportion of gland formation, nuclear pleomorphism, and mitotic count to determine the overall grade of the tumor. The score ranges for grades I, II, and III were 3-5, 6-7, and 8-9, respectively. Tumor staging was performed using the tumor node metastasis (TNM) classification system following the guidelines of the 8th edition of the American Joint Committee on Cancer. TNM stage was classified as I (T_1_N_0_M_0_, T_0~1_N_1mi_M_0_), II (T_0~1_N_1_M_0_, T_2_N_0~1_M_0_, T_3_N_0_M_0_), III (T_0~2_N_2_M_0_, T_3_N_1~2_M_0_, T_4_N_0~2_M_0_, T_0~4_N_3_M_0_), and IV(T_0~4_N_0~3_M_1_).

### Outcome and follow-up

Cancer recurrence and death were study outcomes. The follow-up interval was set as six months and data was collected from clinic visits, telephonic interviews, as well as hospital records. The diagnosis of BC recurrence relied on biopsy, bone scanning, as well as CT/MRI. Information about all-cause deaths was gathered from both patients and their caregivers. Disease-free survival (DFS) was defined as the period from surgery to cancer recurrence or death. Overall survival (OS) was defined as the interval from surgery to death.

### IHC detection and scoring

The expression of CD155, TIGIT, CD96, and CD226 on the membrane of TC and stromal TILs was detected by IHC using the EnVision two-step method. Stromal area was demarcated as the region falling within the boundaries of the invasive tumor. Areas featuring crush artifacts, necrosis, regressive hyalinization, and the biopsy site were excluded from this definition. Scored cells comprised mononuclear cells, specifically lymphocytes and plasma cells, while polymorphonuclear leukocytes were excluded. TILs were measured as the average counts in 10 random high-power fields (HPF, ×400) on IHC sections.

Monoclonal antibody against CD155 (rabbit anti-human, #81254S) were purchased from Cell Signaling Technology Co. Ltd. Monoclonal antibodies against CD226 (rabbit anti-human, #ab2120772) and TIGIT (rabbit anti-human, #ab243903) were purchased from Abcam Co. Ltd. A polyclonal antibody against CD96 (rabbit anti-human, #PA5-97568) was purchased from Invitrogen Co. Ltd. Monoclonal antibodies against PD-L1 (rabbit anti-human, #SP142) were purchased from Roche Shanghai Co. Ltd. Monoclonal antibody against PD-1 (mouse anti-human, # UMAB199) and secondary antibodies were purchased from Beijing Zhongshanjinqiao Biotechnology Co. Ltd.

Positive expression was recorded by brown staining of the cells. PD-L1 positive expression was denoted by the appearance of brown staining in the cytoplasm and/or cell membrane of both immune cells and TC. PD-1 positive expression was manifested as brown-stained cytoplasm within immune cells. Positive expression of CD155/TIGIT/CD96/CD226 on TC in the tumor microenvironment (TME) was defined as brown staining on the cytomembrane. Positive expression of TIGIT/CD96/CD226 in stromal TILs was defined as brown cytoplasmic staining. The expression of CD155, TIGIT, CD96, and CD226 on TC was evaluated by integrating staining intensity and the proportion of positive cells: the proportion of positive TC was categorized into 4 grade based on percentage (grade 0 = 0% positive cells, grade 1 = <1/3 positive cells, grade 2 = 1/3-2/3 positive cells, grade 3 = >2/3 positive cells), and staining intensity was scored as 0 (negative), 1 (weak and incomplete cytomembrane staining), 2 (weak and complete or strong and incomplete cytomembrane staining), or 3 (strong and complete cytomembrane staining); a composite score, with a total range of 0-9, was calculated by multiplying the proportion grade by the corresponding intensity score for all positive TC (i.e., composite score = proportion grade × intensity score).The percentage grade of positive TC indicated the proportion category of TC with molecular expression in the whole section, and the percentage of stromal TILs indicated the proportion of stromal TILs with positive molecule expression in the whole section. High expression of CD155, TIGIT, CD96, and CD226 was defined as a composite score of more than 3 for TC. Because the staining intensity on TILs could not be determined, high stromal expression was defined as more than 2/3 positive cells. Two pathologists estimated the IHC scoring, and a third, higher-level pathologist re-evaluated inconsistent estimations between the two pathologists.

### Statistical analyses

R version 4.3.1 was used to conduct all statistical analyses. Differences between groups were analyzed using Pearson’s chi-square or Fisher’s exact test. The influence of missing data was eliminated during the analysis. Survival curves were plotted using the Kaplan-Meier method, and differences between groups were evaluated using the log-rank test. Cox proportional hazards regression models were established to control for confounding factors. The analysis steps were as follows: ①clinical factors related to prognosis and molecular expression were screened in univariate analysis; ②the selected relevant factors (*p* < 0.10) were adjusted in combination with CD155-TIGIT/CD96/CD226 molecular expression levels, and the relationship between each molecular expression and prognosis was estimated using the Cox multivariate model, with the estimation of hazard ratio (HR) and 95% Confidence interval (CI). All analyses were two-sided and the significance level was set at 0.05.

## Results

### Expression of CD155 and TIGIT/CD96/CD226 on TME TC and stromal TILs

**Figure 1:**
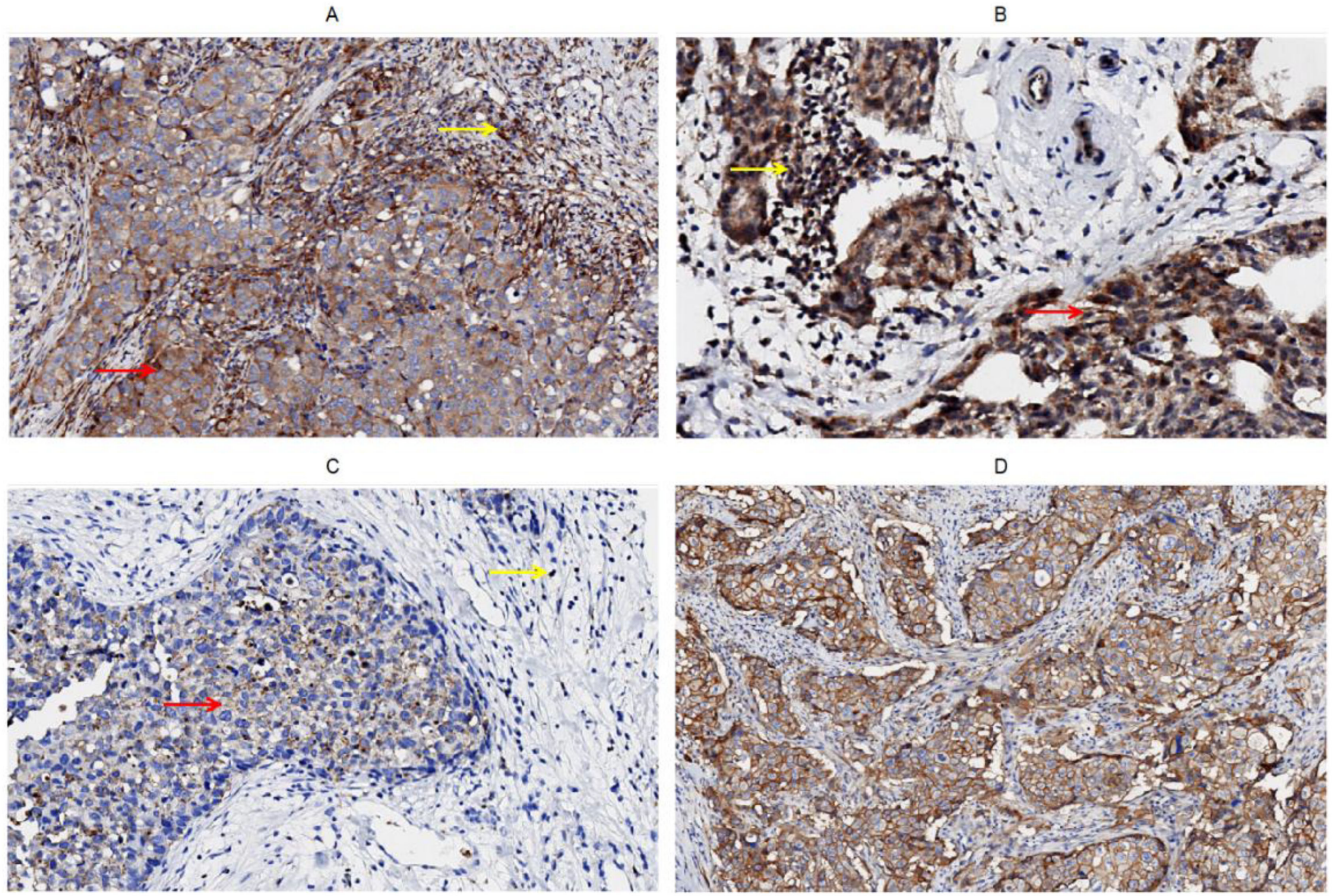
Immunohistochemical staining of CD226, CD96, TIGIT, CD155 on tumor cells and stromal TILs in tumor microenvironment of breast cancer, **(A)** CD226, **(B)** CD96, **(C)** TIGIT, **(D)** CD155.

Both stromal TILs and TC expressed CD226, CD96, and TIGIT (**Figures 1A–C**), but only TC expressed CD155 (**Figure 1D**). Among them, 37.2%, 25.6%, and 17.1% of the patients had more than 2/3 of stromal TILs expressing CD226, CD96, and TIGIT, respectively, and 24.9%,26.9%,84.8% and 56.7% of the patients had high expression levels of CD155, CD226, CD96, and TIGIT in TC, respectively (**Table 1**).

**Table 1:** CD155-CD226/CD96/TIGIT expression in breast cancer.

TME cells	Biomarker expression	N (%)
Stromal TILs	CD226	
	Low	123 (62.8)
	High	73 (37.2)
	CD96	
	Low	154 (74.4)
	High	53 (25.6)
	TIGIT	
	Low	180 (82.9)
	High	37 (17.1)
TC	CD155	
	Low	160 (75.1)
	High	53 (24.9)
	CD226	
	Low	152 (73.1)
	High	56 (26.9)
	CD96	
	Low	31 (15.2)
	High	173 (84.8)
	TIGIT	
	Low	91 (43.3)
	High	119 (56.7)

TILs, tumor-infiltrating lymphocytes; TC, tumor cells

### Correlation between clinicopathological characteristics and CD155-CD226/TIGIT/CD96

High CD226 expression in stromal TILs was associated with ER(*p* = 0.006), PR(*p* = 0.041), and PD-1(*p* = 0.012, **Table 2**). High CD226 expression in TC correlated with low HER-2 expression (*p* = 0.050, **Table 2**). More TNBC patients had high expression of CD96 in stromal TILs (*p* = 0.044, **Table 2**), which was significantly associated with PD-L1 expression in stromal TILs (*p* = 0.012, **Table 2**). High CD96 expression on TC correlated with increased TILs in the TME (*p* = 0.002, **Table 2**). High expression of TIGIT in stromal TILs was correlated with low PR (*p* = 0.025, **Table 2**) but high PD-L1 expression in stromal TILs (*p* = 0.014, **Table 2**). High TIGIT expression in TC was correlated with lower histological grade (*p* = 0.004, **Table 2**). High expression of CD155 was associated with higher TILs levels (*p* = 0.033, **Table 2**), higher proportion of histological grade (*p* = 0.023, **Table 2**), higher Ki-67 index (*p* < 0.001, **Table 2**), higher proportion of TNBC (*p* = 0.020, **Table 2**), and PD-L1 expression on stromal TILs (*p* = 0.040, **Table 2**).

**Table 2:** Correlations between clinicopathological factors and CD155/CD226/TIGIT/CD96 expression.

Characteristic		Expression of CD226 on stromal TILs											Expression of CD226 on TC									Expression of CD96 on stromal TILs							Expression of CD96 on TC					
		Low			High		*χ* ^2^			*p*			Low			High		*χ* ^2^			*p*	Low		High		*χ* ^2^		*p*	Low		High		*χ* ^2^	*p*
		(n=123)			(n=73)								(n=152)			(n=56)						(n=154)		(n=53)					(n=31)		(n=173)			
age																																		
<60		76 (61.8)			48 (65.8)		0.163			0.687			96 (63.2)			37 (66.1)		0.051			0.822	100 (64.9)		35 (66.0)		0		1	17 (54.8)		117 (67.6)		1.383	0.240
≥60		47 (38.2)			25 (34.2)								56 (36.8)			19 (33.9)						54 (35.1)		18 (34.0)					14 (45.2)		56 (32.4)			
TILs																																		
Low		28 (26.7)			20 (27.4)		0			1			33 (24.6)			16 (28.6)		0.148			0.700	38 (27.3)		11 (22.0)		0.303		0.582	15 (50.0)		33 (21.2)		9.480	0.002
High		77 (73.30)			53 (72.6)								101 (75.4)			40 (71.4)						101 (72.7)		39 (78.0)					15 (50.0)		123 (78.8)			
Missing		18			0								18			0						15		3					1		17			
Histology																																		
Ductal		108 (87.8)			66 (90.4)		0.105			0.745			136 (89.5)			50 (89.3)		0			1	139 (90.3)		46 (86.8)		0.201		0.654	28 (90.3)		154 (89.0)		0	1
Lobular/Other		15 (12.2)			7 (9.6)								16 (10.5)			6 (10.7)						15 (9.7)		7 (13.2)					3 (9.7)		19 (11.0)			
Histological grade																																		
I/II		73 (63.5)			41 (62.1)		0.001			0.982			84 (60.4)			38 (73.1)		2.103			0.147	90 (64.3)		31 (62.0)		0.014		0.907	19 (67.9)		101 (63.5)		0.052	0.820
III		42 (36.5)			25 (37.9)								55 (39.6)			14 (26.9)						50 (35.7)		19 (38.0)					9 (32.1)		58 (36.5)			
Missing		8			7								13			4						14		3					3		14			
ER status																																		
Negative		94 (77.7)			65 (94.2)		7.612			0.006			124 (84.4)			47 (85.5)		0			1	125 (83.3)		47 (92.2)		1.739		0.187	24 (80.0)		146 (86.4)		0.401	0.526
Positive		27 (22.3)			4 (5.8)								23 (15.6)			8 (14.5)						25 (16.7)		4 (7.8)					6 (20.0)		23 (13.6)			
Missing		2			4								5			1						4		2					1		4			
PR status																																		
Negative		100 (82.6)			65 (94.2)		4.176			0.041			127 (86.4)			50 (90.9)		0.394			0.530	130 (86.7)		47 (92.2)		0.631		0.427	26 (86.7)		149 (88.2)		0	1
Positive		21 (17.4)			4 (5.8)								20 (13.6)			5 (9.1)						20 (13.3)		4 (7.8)					4 (13.3)		20 (11.8)			
Missing		2			4								5			1						4		2					1		4			
HER-2 status																																		
Negative		113 (93.4)			59 (85.5)		2.330			0.127			136 (92.5)			45 (81.8)		3.837			0.050	138 (92.0)		43 (84.3)		1.725		0.189	29 (96.7)		150 (88.8)		0.997	0.318
Positive		8 (6.6)			10 (14.5)								11 (7.5)			10 (18.2)						12 (8.0)		8 (15.7)					1 (3.3)		19 (11.2)			
Missing		2			4								5			1						4		2					1		4			
TNM stage																																		
I		26 (23.2)			20 (27.4)		4.964			0.174			37 (26.1)			10 (18.2)		3.340			0.342	41 (28.3)		7 (13.7)		5.642		0.130	6 (20.0)		41 (25.0)		6.445	0.092
II		62 (55.4)			35 (47.9)								75 (52.8)			31 (56.4)						71 (49.0)		33 (64.7)					15 (50.0)		88 (53.7)			
III		20 (17.9)			10 (13.7)								23 (16.2)			8 (14.5)						25 (17.2)		7 (13.7)					9 (30.0)		23 (14.0)			
IV		4 (3.6)			8 (11.0)								7 (4.9)			6 (10.9)						8 (5.5)		4 (7.8)					0 (0)		12 (7.3)			
Missing		11			0								10			1						9		2					1		9			
PD-1																																		
<10%		13 (12.6)			2 (3.0)		8.907			0.012			13 (10.1)			3 (6.8)		0.670			0.715	14 (10.9)		1 (2.3)		3.730		0.155	4 (14.8)		11 (7.7)		1.839	0.399
10%-50%		67 (65.0)			57 (85.1)								92 (71.3)			34 (77.3)						89 (69.5)		36 (81.8)					17 (63.0)		105 (73.9)			
≥50%		23 (22.3)			8 (11.9)								24 (18.6)			7 (15.9)						25 (19.5)		7 (15.9)					6 (22.2)		26 (18.3)			
Missing		20			6								23			12						26		9					4		31			
Ki-67 index																																		
<14%		23 (19.0)			16 (23.5)		0.302			0.582			30 (20.4)			10 (18.2)		0.024			0.877	34 (23.0)		8 (15.4)		0.917		0.338	9 (30.0)		32 (19.0)		1.252	0.263
≥14%		98 (81.0)			52 (76.5)								117 (79.6)			45 (81.8)						114 (77.0)		44 (84.6)					21 (70.0)		136 (81.0)			
Missing		2			5								5			1						6		1					1		5			
Molecular type																																		
Non-TNBC		62 (52.1)			31 (46.3)		0.373			0.541			67 (46.5)			26 (48.1)		0.002			0.965	75 (51.0)		17 (33.3)		4.077		0.044	17 (56.7)		73 (44.0)		1.176	0.278
TNBC		57 (47.9)			36 (53.7)								77 (53.5)			28 (51.9)						72 (49.0)		34 (66.7)					13 (43.3)		93 (56.0)			
Missing		4			6								8			2						7		2					1		7			
PD-L1 (tumor cells)																																		
<1%		73 (70.9)			49 (75.4)		0.213			0.645			90 (69.8)			35 (83.3)		2.316			0.128	97 (77.0)		28 (62.2)		2.962		0.085	20 (80.0)		103 (71.5)		0.403	0.525
≥1%		30 (29.1)			16 (24.6)								39 (30.2)			7 (16.7)						29 (23.0)		17 (37.8)					5 (20.0)		41 (28.5)			
Missing		20			8								23			14						28		8					6		29			
PD-L1(stromal cells)																																		
<1%		68 (66.0)			35 (53.8)		2.003			0.157			75 (58.6)			28 (65.1)		0.332			0.565	82 (65.1)		19 (42.2)		6.251		0.012	18 (72.0)		81 (56.3)		1.577	0.209
≥1%		35 (34.0)			30 (46.2)								53 (41.4)			15 (34.9)						44 (34.9)		26 (57.8)					7 (28.0)		63 (43.7)			
Missing		20			8								24			13						28		8					6		29			
Adjuvant radiotherapy																																		
No		80 (72.7)			43 (61.4)		2.029			0.154			101 (71.6)			31 (59.6)		2.012			0.156	102 (71.3)		27 (55.1)		3.654		0.056	16 (55.2)		110 (68.8)		1.471	0.225
Yes		30 (27.3)			27 (38.6)								40 (28.4)			21 (40.4)						41 (28.7)		22 (44.9)					13 (44.8)		50 (31.3)			
Missing		13			3								11			4						11		4					2		13			
Adjuvant chemotherapy																																		
No		17 (16.5)			15 (24.6)		1.121			0.290			25 (19.4)			9 (18.8)		0			1	27 (20.9)		8 (17.0)		0.131		0.718	7 (28.0)		27 (18.2)		0.745	0.388
Yes		86 (83.5)			46 (75.4)								104 (80.6)			39 (81.3)						102 (79.1)		39 (83.0)					18 (72.0)		121 (81.8)			
Missing		20			12								23			8						25		6					6		25			
Adjuvant endocrine therapy																																		
No		64 (58.7)			45 (62.5)		0.125			0.723			85 (61.2)			35 (67.3)		0.379			0.538	84 (59.2)		35 (71.4)		1.843		0.175	13 (48.1)		106 (65.8)		2.400	0.121
Yes		45 (41.3)			27 (37.5)								54 (38.8)			17 (32.7)						58 (40.8)		14 (28.6)					14 (51.9)		55 (34.2)			
Missing		14			1								13			4						12		4					4		12			
Adjuvant targeted therapy																																		
No		113 (96.6)			68 (95.8)		0			1				139 (95.9)		53 (98.1)		0.120			0.730	144 (97.3)		48 (94.1)		0.387		0.534	29 (100.0)		160 (95.8)		0.337	0.561
Yes		4 (3.4)			3 (4.2)									6 (4.1)		1 (1.9)						4 (2.7)		3 (5.9)					0 (0)		7 (4.2)			
Missing		6			2									7		2						6		2					2		6			

Characteristic	Expression of TIGIT on stromal TILs				Expression of TIGIT on TC				CD155			
	Low	High	*χ* ^2^	*p*	Low	High	*χ* ^2^	*p*	Low	High	*χ* ^2^	*p*
	(n=180)	(n=37)			(n=91)	(n=119)			(n=160)	(n=53)		
Age												
<60	114 (63.3)	27 (73.0)	0.865	0.352	62 (68.1)	76 (63.9)	0.249	0.618	104 (65.0)	36 (67.9)	0.049	0.824
≥60	66 (36.7)	10 (27.0)			29 (31.9)	43 (36.1)			56 (35.0)	17 (32.1)		
TILs												
Low	44 (27.3)	8 (21.6)	0.254	0.614	22 (30.1)	29 (24.6)	0.457	0.499	43 (27.7)	4 (10.0)	4.544	0.033
High	117 (72.7)	29 (78.4)			51 (69.9)	89 (75.4)			112 (72.3)	36 (90.0)		
Missing	19	0			18	1			5	13		
Histology												
Ductal	160 (88.9)	34 (91.9)	0.061	0.805	84 (92.3)	105 (88.2)	0.552	0.458	137 (85.6)	51 (96.2)	3.356	0.067
Lobular/Other	20 (11.1)	3 (8.1)			7 (7.7)	14 (11.8)			23 (14.4)	2 (3.8)		
Histological grade												
I/II	105 (64.0)	22 (64.7)	0	1	44 (53.0)	81 (74.3)	8.495	0.004	98 (69.0)	26 (50.0)	5.171	0.023
III	59 (36.0)	12 (35.3)			39 (47.0)	28 (25.7)			44 (31.0)	26 (50.0)		
Missing	16	3			8	10			18	1		
ER status												
Negative	145 (83.3)	36 (97.3)	3.800	0.051	72 (81.8)	102 (87.9)	1.043	0.307	132 (84.6)	45 (88.2)	0.167	0.683
Positive	29 (16.7)	1 (2.7)			16 (18.2)	14 (12.1)			24 (15.4)	6 (11.9)		
Missing	6	0			3	3			4	2		
PR status												
Negative	148 (85.1)	37 (100)	4.999	0.025	73 (83.0)	105 (90.5)	1.938	0.164	136 (87.2)	47 (92.2)	0.507	0.477
Positive	26 (14.9)	0 (0)			15 (17.0)	11 (9.5)			20 (12.8)	4 (7.8)		
Missing	6	0			3	3			4	2		
HER-2 status												
Negative	158 (90.8)	32 (86.5)	0.244	0.621	84 (95.5)	101 (87.1)	3.232	0.072	139 (89.1)	46 (90.2)	0	1
Positive	16 (9.2)	5 (13.5)			4 (4.5)	15 (12.9)			17 (10.9)	5 (9.8)		
Missing	6	0			3	3			4	2		
TNM stage												
I	40 (24.0)	10 (27.0)	0.205	0.977	23 (28.0)	26 (22.2)	1.729	0.631	36 (22.9)	12 (26.1)	6.427	0.093
II	89 (53.3)	19 (51.4)			41 (50.0)	65 (55.6)			92 (58.6)	20 (43.5)		
III	27 (16.2)	6 (16.2)			14 (17.1)	17 (14.5)			19 (12.1)	12 (26.1)		
IV	11 (6.6)	2 (5.4)			4 (4.9)	9 (7.7)			10 (6.4)	2 (4.3)		
Missing	13	0			9	2			3	7		
PD-1												
<10%	14 (9.7)	2 (6.3)	1.432	0.489	10 (14.5)	5 (4.9)	5.127	0.077	14 (10.1)	3 (8.1)	0.188	0.910
10%-50%	102 (70.8)	26 (81.3)			46 (66.7)	80 (77.7)			98 (70.5)	26 (70.3)		
≥50%	28 (19.4)	4 (12.5)			13 (18.8)	18 (17.5)			27 (19.4)	8 (21.6)		
Missing	36	5			22	16			21	16		
Ki-67 index												
<14%	36 (20.8)	8 (21.6)	0	1	17 (19.3)	24 (20.7)	0.004	0.948	42 (27.3)	1 (1.9)	13.629	<0.001
≥14%	137 (79.2)	29 (78.4)			71 (80.7)	92 (79.3)			112 (72.7)	51 (98.1)		
Missing	7	0			3	3			6	1		
Molecular type												
Non-TNBC	82 (48.0)	13 (36.1)	1.236	0.266	33 (38.4)	58 (50.9)	2.608	0.106	81 (52.3)	16 (32.0)	5.438	0.020
TNBC	89 (52.0)	23 (63.9)			53 (61.6)	56 (49.1)			74 (47.7)	34 (68.0)		
Missing	9	1			5	5			5	3		
PD-L1 (tumor cells)												
<1%	104 (72.2)	24 (77.4)	0.136	0.712	48 (65.8)	77 (78.6)	2.874	0.090	103 (77.4)	26 (61.9)	3.216	0.073
≥1%	40 (27.8)	7 (22.6)			25 (34.2)	21 (21.4)			30 (22.6)	16 (38.1)		
Missing	36	6			18	21			27	11		
PD-L1 (stromal cells)												
<1%	93 (64.6)	12 (38.7)	6.078	0.014	38 (52.1)	63 (64.3)	2.107	0.147	86 (64.7)	19 (45.2)	4.241	0.040
≥1%	51 (35.4)	19 (61.3)			35 (47.9)	35 (35.7)			47 (35.3)	23 (54.8)		
Missing	36	6			18	21			27	11		
Adjuvant radiotherapy												
No	115 (70.6)	20 (55.6)	2.391	0.122	63 (76.8)	69 (62.2)	4.039	0.045	98 (65.3)	34 (70.8)	0.278	0.598
Yes	48 (29.4)	16 (44.4)			19 (23.2)	42 (37.8)			52 (34.7)	14 (29.2)		
Missing	17	1			9	8			10	5		
Adjuvant chemotherapy												
No	28 (18.4)	9 (28.1)	1.004	0.316	15 (18.5)	18 (18.6)	0	1	29 (21.3)	5 (10.9)	1.833	0.176
Yes	124 (81.6)	23 (71.9)			66 (81.5)	79 (81.4)			107 (78.7)	41 (89.1)		
Missing	28	5			10	22			24	7		
Adjuvant endocrine therapy												
No	103 (63.2)	23 (63.9)	0	1	59 (72.8)	63 (56.8)	4.557	0.033	85 (57.0)	38 (79.2)	6.660	0.010
Yes	60 (36.8)	13 (36.1)			22 (27.2)	48 (43.2)			64 (43.0)	10 (20.8)		
Missing	17	1			10	8			11	5		
Adjuvant targeted therapy												
No	164 (95.9)	36 (100.0)	0.530	0.467	85 (97.7)	108 (95.6)	0.179	0.672	149 (96.8)	47 (94.0)	0.204	0.651
Yes	7 (4.1)	0 (0)			2 (2.3)	5 (4.4)			5 (3.2)	3 (6.0)		
Missing	9	1			4	6			6	3		

### Survival analyses

**Figure 2:**
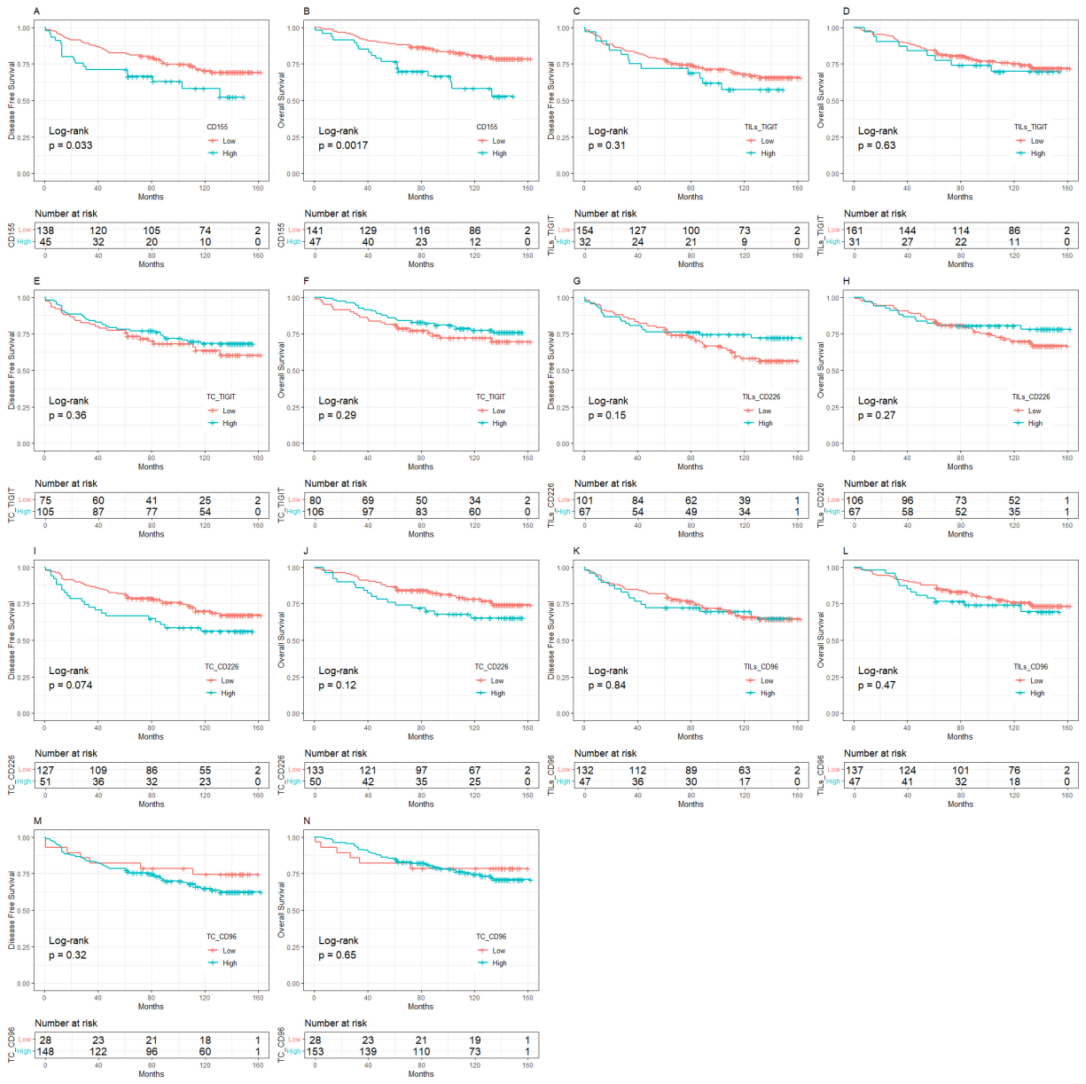
Kaplan-Meier curve of the relationship between molecular expression of CD155-TIGIT/CD226/CD96 and prognosis, **(A)** CD155 expression and DFS, **(B)** CD155 expression and OS, **(C)** TIGIT expression on stromal TILs and DFS, **(D)** TIGIT expression on stromal TILs and OS, **(E)** TIGIT expression on tumor cells and DFS, **(F)** TIGIT expression on tumor cells and OS, **(G)** CD226 expression on stromal TILs and DFS, **(H)** CD226 expression on stromal TILs and OS, **(I)** CD226 expression on tumor cells and DFS, **(J)** CD226 expression on tumor cells and OS, **(K)** CD96 expression on stromal TILs and DFS, **(L)**. CD96 expression on stromal TILs and OS, **(M)**. CD96 expression on tumor cells and DFS, **(N)**. CD96 expression on tumor cells and OS. TILs, Tumor- infiltrating lymphocytes.

**Figure 3:**
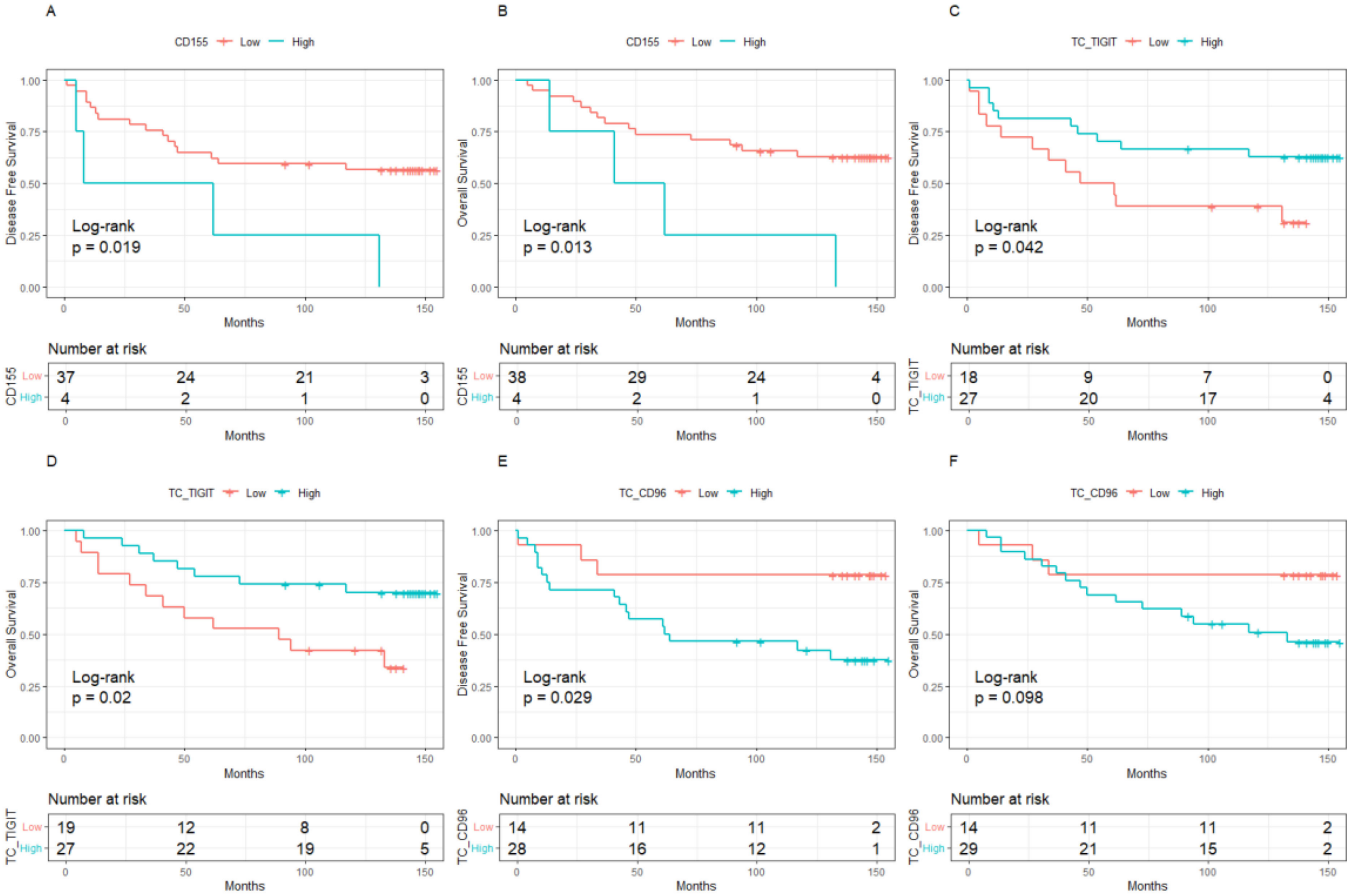
Kaplan-Meier curve of the relationship between molecular expression and prognosis in the population with low level of TILs, **(A)**. CD155 expression and DFS, **(B)**. CD155 expression and OS, **(C)**. TIGIT expression on tumor cells and DFS, **(D)**. TIGIT expression on tumor cells and OS, **(E)**. CD96 expression on tumor cells and DFS, **(F)**. CD96 expression on tumor cells and OS. TILs, Tumor- infiltrating lymphocytes.

**Figure 4:**
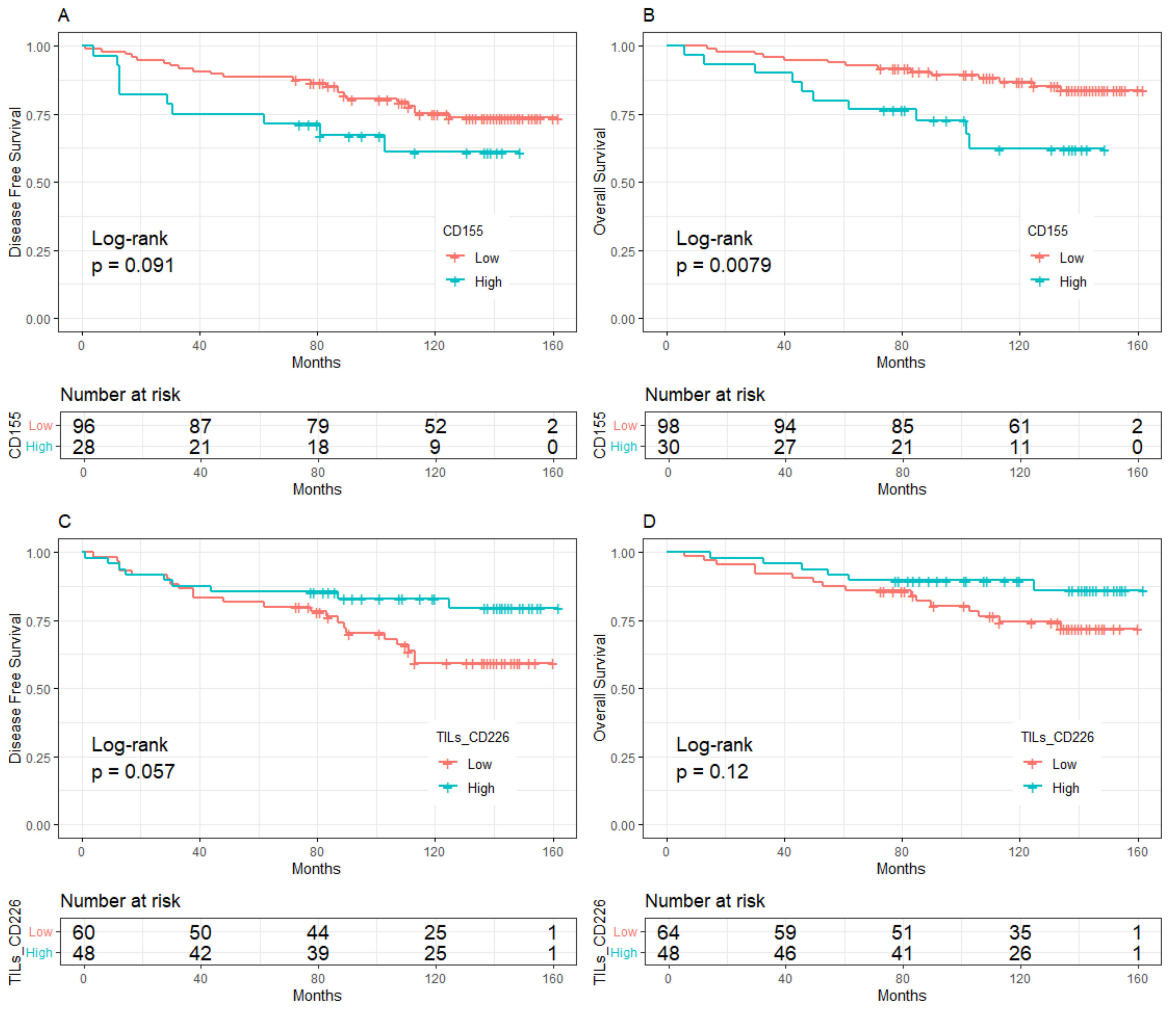
Kaplan-Meier curve of the relationship between molecular expression and prognosis in the population with high level of TILs, **(A)**. CD155 expression and DFS, **(B)**. CD155 expression and OS, **(C)**. CD226 expression on stromal TILs and DFS, **(D)**. CD226 expression on stromal TILs and OS. TILs, Tumor- infiltrating lymphocytes.

The median follow-up was 10 years (95%CI:8.8-11.0). BC patients with low expression of CD155 had 10-year DFS and OS rates of 70.37% and 80.64%, respectively, which were significantly higher than those with high expression of CD155 (58.81% for DFS, *p* = 0.033; 58.24% for OS, *p* = 0.002, **Figure 2**). Among patients with low TME infiltration of stromal TILs, the 10-year DFS and OS rates differed among different biomarker expression groups. For CD155, the rates were 56.49% and 62.70% in the low-expression group and 25.00% (*p* = 0.019) and 25.00% (*p* = 0.013) in the high-expression group, respectively (**Figure 3**). Regarding TC CD96, the low expression group of patients had rates of 78.57% and 78.57%, while the high expression group of patients had rates of 42.21% (*p* = 0.029) and 50.73% (*p* = 0.098), respectively (**Figure 3**). Patients with low TIGIT expression on TC had 10-year DFS and OS rates of 38.89% and 42.11%, respectively, and the high expression group had 62.75% (*p* = 0.042) and 69.96% (*p* = 0.020), respectively (**Figure 3**). In patients with high TME infiltration of stromal TILs, patients with low expression of CD155 had 10-year DFS and OS rates of 75.19% and 86.89%, respectively, compared with 61.12% (*p* = 0.091) and 62.26% (*p* = 0.008) among those with high expression of CD155, respectively (**Figure 4**). Additionally, patients with low expression of CD226 on stromal TILs had 10-year DFS and OS rates of 59.23% and 74.38%, respectively, whereas the 10-year rates of DFS and OS were 83.04% (*p* = 0.057) and 89.58% (*p* = 0.120) among patients with high expression, respectively (**Figure 4**).

**Figure 5:**
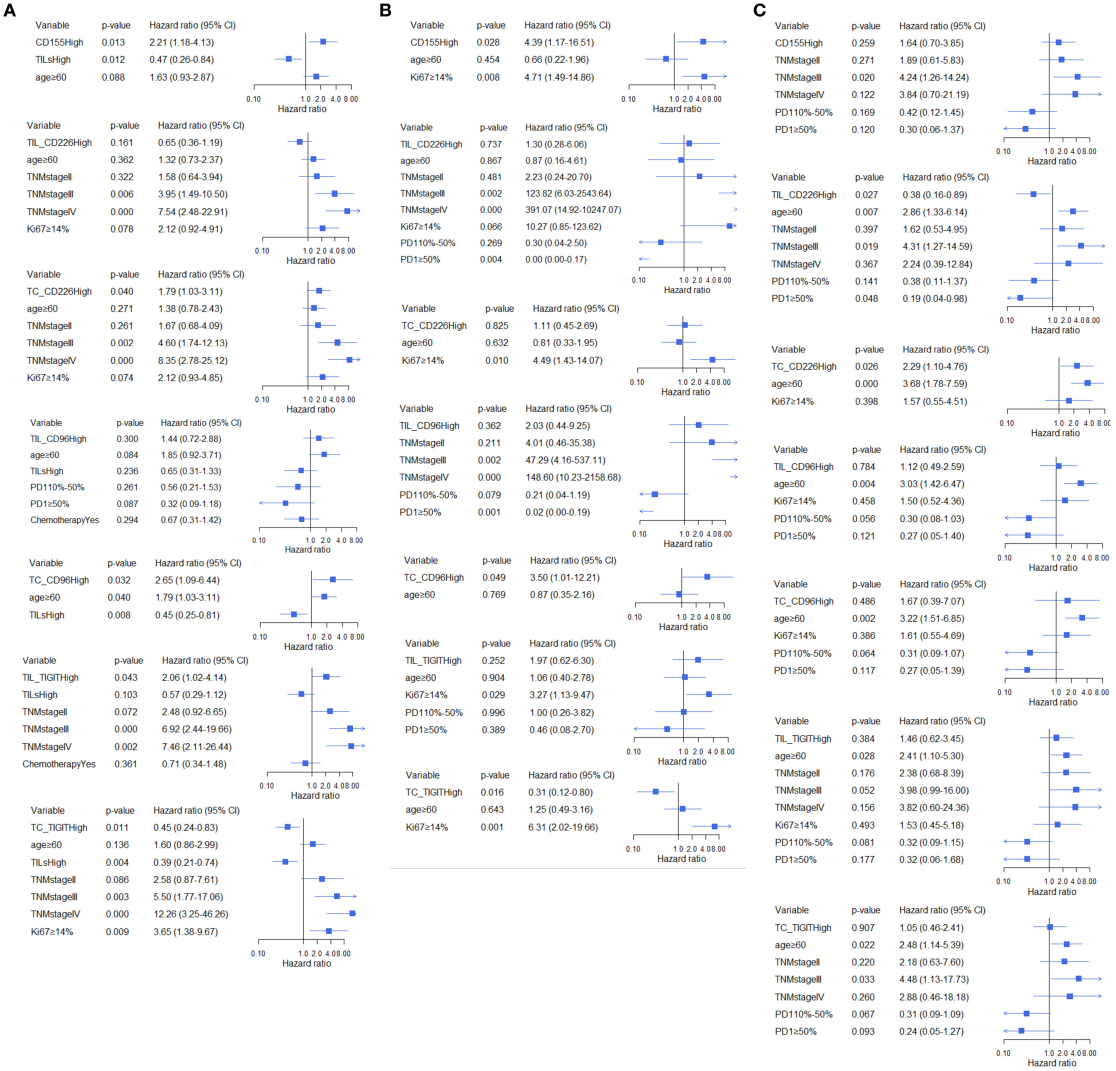
Multivariate analysis of the association between CD155, CD226, TIGIT, and CD96 and DFS across different TILs subgroups, **(A)** total participants, **(B)** subgroup with low TME infiltration of TILs, **(C)** subgroup with high TME infiltration of TILs.

**Figure 6:**
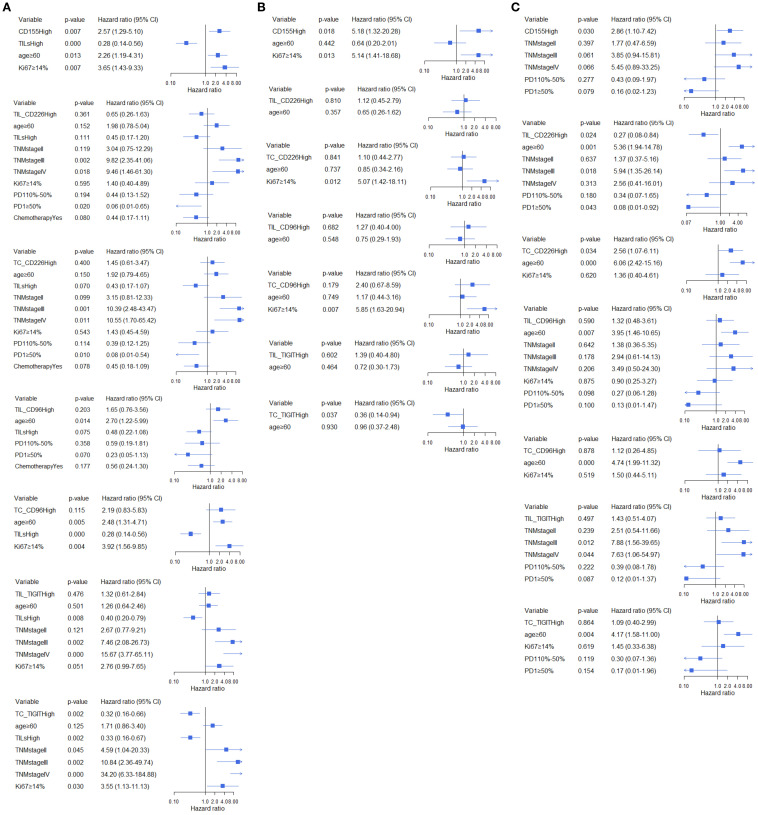
Multivariate analysis of the association between CD155, CD226, TIGIT, and CD96 and OS across different TILs subgroups, **(A)** total participants, **(B)** subgroup with low TME infiltration of TILs, **(C)** subgroup with high TME infiltration of TILs.

In all BC patients, high expression of CD155 increased 2.21-fold the risk of relapse (95%CI:1.18-4.13, **Figure 5A**) and a 2.57-fold higher risk of death (95%CI:1.29-5.10, **Figure 6A**). High expression of TC CD226 (HR = 1.79, 95%CI: 1.03-3.11), TC CD96 (HR = 2.65, 95%CI:1.09-6.44), and TIGIT on stromal TILs (HR = 2.06, 95%CI:1.02-4.14) were associated with an increased risk of relapse (**Figure 5A**). However, high expression of TC TIGIT was associated with a 55% reduction in relapse risk (HR = 0.45, 95%CI:0.24-0.83, **Figure 5A**) and a 68% reduction in death risk (HR = 0.32, 95%CI:0.16-0.66, **Figure 6A**). Among patients with low TME infiltration of stromal TILs, high expression of CD155 was associated with poor DFS (HR = 4.39, 95%CI:1.17-16.51, **Figure 5B**) and OS (HR = 5.18, 95%CI:1.32-20.28, **Figure 6B**). High TC CD96 expression (HR = 3.50, 95%CI: 1.01-12.21, **Figure 5B**) was associated with unfavorable DFS, while high TC TIGIT expression was associated with favorable DFS (HR = 0.31, 95%CI:0.12-0.80, **Figure 5B**) and OS (HR = 0.36, 95%CI: 0.14-0.94, **Figure 6B**). In patients with high TME infiltration of stromal TILs, high expression of CD155 was associated with a 2.86-fold higher risk of death (HR = 2.86, 95%CI:1.10-7.42, **Figure 6C**) and high expression of TC CD226 was associated with a 2.29-fold high risk of relapse (HR = 2.29, 95%CI:1.10-4.76, **Figure 5C**) and a 2.56-fold higher risk of death (HR = 2.56, 95%CI: 1.07-6.11, **Figure 6C**), while high expression of CD226 on stromal TILs was associated with a favorable DFS (HR = 0.38, 95%CI:0.16-0.89, **Figure 5C**) and OS (HR = 0.27, 95%CI:0.08-0.84, **Figure 6C**).

## Discussion

In this study, CD155 was detected in BC TME TC, and CD226/TIGIT/CD96 was observed to be expressed on both BC stromal TILs and TME TC. The four immune checkpoint molecules were systematically evaluated for the first time to explore their association with the clinical characteristics and prognosis of patients with BC. Previous studies had confirmed the survival benefit of TILs and even the prediction of the response to neoadjuvant chemotherapy (17, 18). TILs had been reported to be capable of guide treatment decisions (19, 20), including ICI immunotherapy (21). The expression of CD155-TIGIT/CD226/CD96 on TC and TILs interacted with TME TILs to exhibit diverse prognostic effects on BC.

Similarly, many studies had reported the upregulation of CD155 and its correlation with age, disease stage, tumor size, molecular subtype, and other clinical characteristics of BC patients (22–24). The unfavorable prognostic effect of high CD155 expression was consistent with that reported by Yong et al. (25), Li et al. (16), Song (26)., Stamm H et al. (27), and Triki H et al. (28). Furthermore, a meta-analysis conducted by Zhang et al. (29) showed an unfavorable effect of CD155 on BC prognosis (pooled HR = 2.137, 95%CI:1.448-3.154). Additionally, CD155 had been confirmed to be linked to the invasion and migration of BC cells, and its downregulation could induce apoptosis of BC cells (30). A recent study demonstrated that fucosylated-CD155 secreted by brain metastasis-associated fibroblasts might regulate intercellular junctions and actin cytoskeleton signaling to enhance BC invasion (31). Asynchronous blocking of PD-L1 and CD155 with polymer nanoparticles had been found to inhibit the progression and metastasis of TNBC (32). CD155 had great potential as a novel immunotherapeutic target in BC.

TIGIT is mainly expressed on immune cells, directly inhibits the immune response by activating immune cells, and indirectly inhibits the anti-tumor response by binding to CD155 (8). This study indicated that TIGIT expression was upregulated in TME stromal TILs and TC in 17.1% and 56.7% of BC patients, respectively, which was consistent with the results found by Tang (33). This study discovered that BC patients with high TIGIT expression in TILs had shorter DFS than those with low TIGIT expression. However, Xie et al. (34) observed that the relationship between TIGIT expression in immune cells and OS was not statistically significant. The main reason for this disparity might be the small sample size of their study. In contrast, Boissiere-Michot et al. (35) considered that high TIGIT expression in stromal cells was associated with longer prognosis in non-molecular apocrine TNBC, possibly because TIGIT had different effects on prognosis in different molecular subtypes. It was necessary to further explore the prognostic value of TIGIT in BC from multiple perspectives to achieve personalized management in the future. In a study conducted by Luo (36). showed that the upregulation of TIGIT expression in cancer tissues among patients with invasive BC, had a trend of good prognosis, although the trend was not statistically significant. Although Song (26). confirmed that upregulated expression of TIGIT in BC tissues was related to poor prognosis (DFS: HR = 5.199, 95%CI:1.477-18.292), Zhang et al. (37) indicated no statistically significant (DFS: HR = 1.110, 95%CI:0.492-2.502) relationship between high TIGIT expression on TC and poor prognosis in TNBC. The inconsistent results observed between studies were related to the variable expression site of TILs or TC. TC-expressing TIGIT indicated a favorable prognosis, but TIL-expressing TIGIT indicated an unfavorable prognosis of BC. TIGIT is a co-inhibitory receptor of CD155 in malignant tumors. Synergistically blocking TIGIT and HIF-1α inhibited the growth and development of BC cells (38). Co-blocking of TIGIT and IL1β activated anti-tumor immunity, inhibited bone metastasis of BC, and improved the survival rate (39). Therefore, upregulation of TIGIT on TILs triggered an immune escape mechanism (40). Similarly, TIGIT might affect the proliferation or inhibit the growth of malignant TC. However, the underlying mechanism needed to be further confirmed.

CD96 was reported to be exclusively expressed on immune cells (9); however, this study verified that CD96 could be detected on both TILs and TC, and 84.8% of patients expressed high levels of CD96 on TC. High expression of CD96 on TC was related to shorter DFS than low expression, especially among patients with low TME infiltration of TILs, in line with the study performed by Li et.al (41). In another study conducted by Xu et.al (42). suggested that high tumoral CD96 expression was associated with a poor prognosis in gastric cancer. The cytoplasmic domain of CD96 contains a short alkaline/proline-rich motif and a single ITIM-like domain with a potential inhibitory function, as well as a YXXM motif with the potential to activate receptors (43). At the same time, bioinformatic analysis performed by Ye et al. (44). exhibited that CD96 played a vital but contradictory role in different cancers. These results indicated that the biological effects of CD96 were not limited to immune cells, and the signal transduction mechanism expressed on TC and immune cells of the BC TME still needed to be further explored.

CD226 is expressed in most tumors, and its high expression is associated with improved clinical outcomes (45). In BC, CD226 was significantly downregulated on the surface of CD56^+^CD16 ^+^NK cells and CD56^+^CD16^-^NK cells (46), and high genetic expression was associated with good prognosis in BC patients with stage II and III, as well as Luminal B (47). However, these data revealed that high CD226 expression in TC and TILs was related to poor and better prognosis, respectively, in the population with more TILs. Nonetheless, two recently published studies showed a favorable prognosis for CD226 on immune cells of gastric cancer (48, 49). CD226 is an activating receptor (50), and its expression on TILs might enhance the anti-tumor ability of TILs (51), whereas its expression on TC contributes to the immune escape ability of TC. The inconsistent prognostic effect of CD226 on stromal TILs and TC required basic studies to explore the underlying mechanism.

This study was the first to evaluate the expression of the CD155-CD226/TIGIT/CD96 protein complex in BC patients and its association with relapse and death. CD155 was reported as an immune checkpoint protein, CD226 as a co-stimulatory receptor of the immune checkpoint axis, and TIGIT and CD96 as co-inhibitory receptors of the immune checkpoint axis. Therefore, CD226 expression on immune cells indicated an increase in immune function and a favorable prognosis, whereas TIGIT expression on immune cells suppressed immune function and was related to an unfavorable prognosis. In contrast, CD226 expression on TC increased immune escape function and an unfavorable prognosis, but TIGIT expression on TC increased cell apoptosis and was associated with a favorable prognosis. CD96 expression on TC seemed to correlate with BC relapse, but the expression on immune cells did not seem to correlate with prognosis. However, these basic mechanisms required further investigation.

## Conclusion

CD155 expression was detected solely on TC, and the receptor expression of CD226/TIGIT/CD96 was detected in both TC and stromal TILs in the BC TME. High expression of CD155 indicates an unfavorable prognosis for BC. However, high expression of CD226/TIGIT/CD96 had diverse effects on BC prognosis, CD226 expression on TILs and TIGIT expression on TC correlated with favorable prognosis, and CD226 and CD96 expression on TC and TIGIT expression on TILs may be related to unfavorable prognosis. CD155-CD226/TIGIT/CD96 should be evaluated as a whole complex of immune checkpoint axis molecules to assess prognosis completely. For the diverse effect on prognosis, ICIs targeting the complex axis should check the tumoral and immune expression CD155-CD226/TIGIT/CD96 together.

## Data Availability

The raw data supporting the conclusions of this article will be made available by the authors, without undue reservation.
